# Case report: Villaret's syndrome caused by middle ear adenocarcinoma in a cat

**DOI:** 10.3389/fvets.2023.1225567

**Published:** 2023-07-27

**Authors:** Dong-Jae Kang, Won-Keun Park, So-Yeon Kim, Dong-Hoon Shin, Hee-Myung Park, Min-Hee Kang

**Affiliations:** ^1^Department of Veterinary Internal Medicine, College of Veterinary Medicine, Konkuk University, Seoul, Republic of Korea; ^2^Yonggang Animal Hospital, Seoul, Republic of Korea; ^3^Department of Bio-animal Care, Jangan University, Hwaseong, Gyeonggi-do, Republic of Korea

**Keywords:** cat, Villaret's syndrome, adenocarcinoma, middle ear, lower cranial nerve dysfunction

## Abstract

A 7-year-old castrated male American Shorthair cat presented with left-side Horner's syndrome and voice change. The overall clinical presentation included dysphagia, intermittent coughing, unilateral miosis, and third eyelid protrusion of the left eye. A topical 1% phenylephrine was applied, and miosis and protrusion of the third eyelid disappeared within 20 min which suggested a post-ganglionic lesion. Laryngoscopy showed left-sided laryngeal paralysis. Computed tomography (CT) identified a mass lesion invading outside of the left tympanic bulla with osteolysis. Endoscopically assisted ventral bulla osteotomy was performed for tumor resection and definitive diagnosis. Middle ear adenocarcinoma was diagnosed based on histopathology. It appears that these neurological signs occurred due to adenocarcinoma in the tympanic bulla, penetrating the jugular foramen and the hypoglossal canal and damaging the cranial nerve IX (glossopharyngeal nerve), X (vagus nerve), XI (accessory nerve), and XII (hypoglossal nerve) and the sympathetic nerve. To the best of our knowledge, this is the first case report of Villaret's syndrome associated with middle ear adenocarcinoma affecting the nerves passing through the jugular foramen and hypoglossal canal in cats.

## Introduction

Villaret's syndrome is a rare neurological disorder that affects the lower cranial nerves (LCNs) and sympathetic nerves, reported in human medicine in 1916 (1). This syndrome is mainly caused by trauma, tumor, or aneurysm in the retroparotid space and causes paralysis of cranial nerves IX, X, XI, and XII and cervical sympathetic nerves (2, 3). Due to the anatomic location of the lesions, multiple LCNs could be affected, and slightly different diseases may occur depending on the affected cranial nerves. Because of the difficulty in assessing the exact anatomy and pathophysiology of the cranial nerves, diseases affecting the LCNs are very rarely reported in veterinary medicine (4).

This report describes a rare feline adenocarcinoma affecting tympanic bulla and emphasizes the importance of an accurate neurological evaluation as well as a detailed clinical assessment. The present report describes Villaret's syndrome for the first time in veterinary medicine.

## Case description

### Case presentation and diagnostic investigations

A 7-year-old male castrated 6.2-kg American Shorthair cat was presented for evaluation of a 3-week history of protrusion of the left third eyelid, voice change, and anorexia. The cat had a history of herpes virus infection as a kitten and was otherwise healthy. The cat lived indoors only. Recent clinical signs assumed respiratory problems and the cat was treated symptomatically, but there was no response and clinical signs gradually worsened.

On physical examination, the cat was slightly depressed but vital signs were within normal limits. The cat showed left-sided third eyelid protrusion, miosis, enophthalmos, and ptosis consistent with Horner's syndrome. The gag reflex was reduced, and abnormal phonation (voice change) was marked. Other physical and neurological examinations were unremarkable.

**Figure 1:**
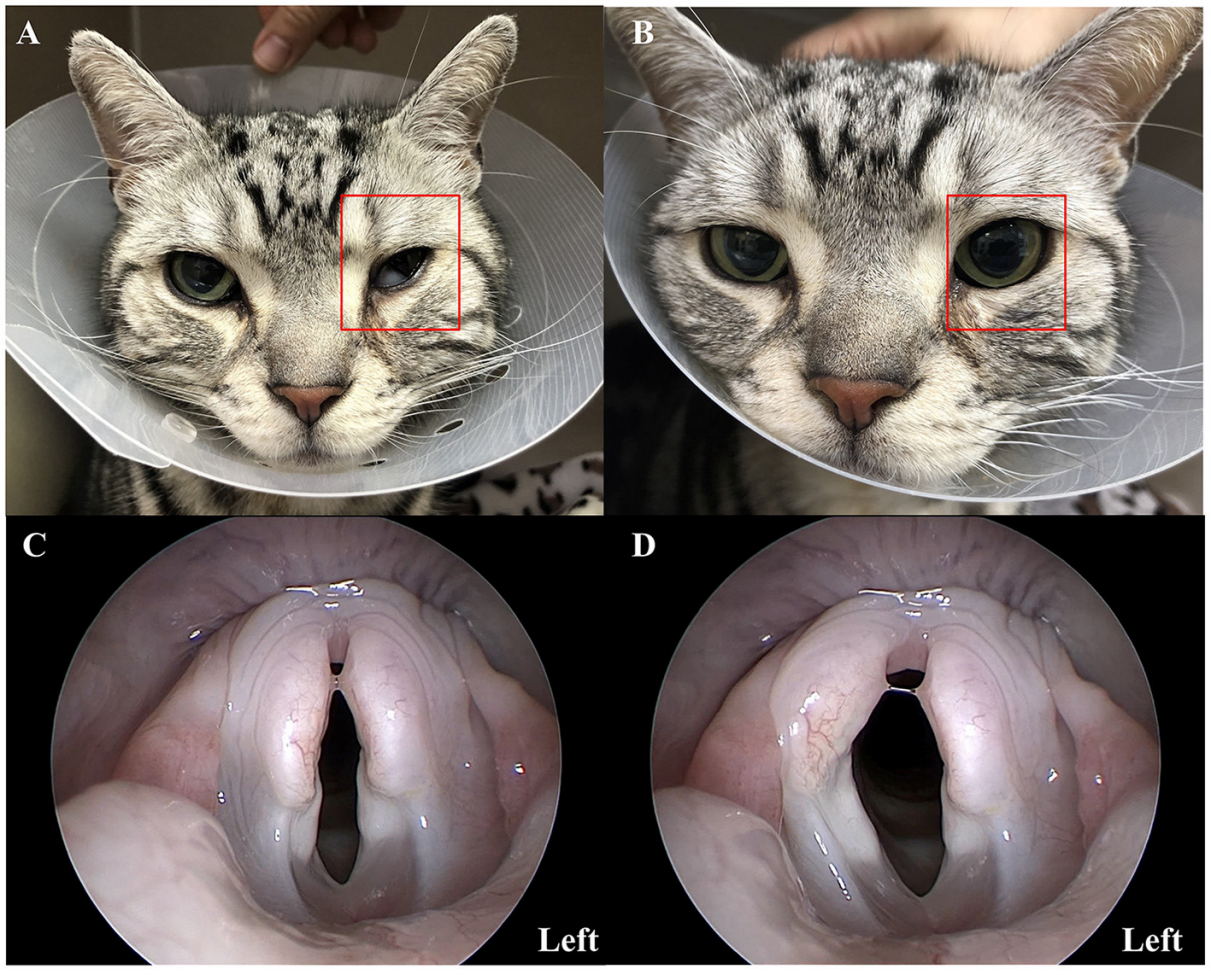
Clinical presentation of a 7-year-old American short hair cat showing signs of Horner's syndrome and laryngeal paralysis. Pharmacological testing with topical 1% phenylephrine **(A, B)**. The left eye of a cat (red rectangle) showed miosis, protrusion of the third eyelid, ptosis, and enophthalmos consistent with Horner's syndrome **(A)**. After topical administration of 1% phenylephrine to the left eye, clinical signs resolved within 20 min **(B)**. Laryngoscopy images **(C, D)** showed the right and left arytenoid cartilage movement during inspiration. It confirmed the left-sided laryngeal paralysis.

Serum biochemistry and complete blood count revealed mild leukocytosis. A topical 1% phenylephrine test was performed for neurolocalization, and the related clinical signs resolved within 20 min after application (Figures 1A, B). Therefore, it is suspected that the lesion is located in the cranial cervical ganglion that originates in the ventromedial to tympanic bulla and extends to the orbit. In addition to Horner's syndrome, the cat also had other neurological deficits (reduced gas reflex) and abnormal phonation, thus, further evaluation of the pharynx, larynx, and middle ear was required.

The cat was premedicated with butorphanol (0.2 mg/kg, intramuscular injection, Myungmoon, Seoul, South Korea). Anesthesia was induced with propofol (6 mg/kg, intravenous injection, Myungmoon, Seoul, South Korea). The cat was positioned in sternal recumbency, and the nasopharynx and larynx were examined. Inspection of the oral cavity, oropharynx, and larynx was performed using laryngoscopy (Veterinary otoscope, 67260OSA, Karl Storz, Tuttlingen, Germany), and inspection of the caudal choanae and nasopharynx was evaluated using retroflex rhinoscopy (feline videoendoscope, 60511 NKS, Karl Storz, Tuttlingen, Germany). Abnormal movements of the larynx consistent with left laryngeal paralysis were confirmed (Figures 1C, D) during the examination. Caudal aberrant turbinate was also identified.

**Figure 2:**
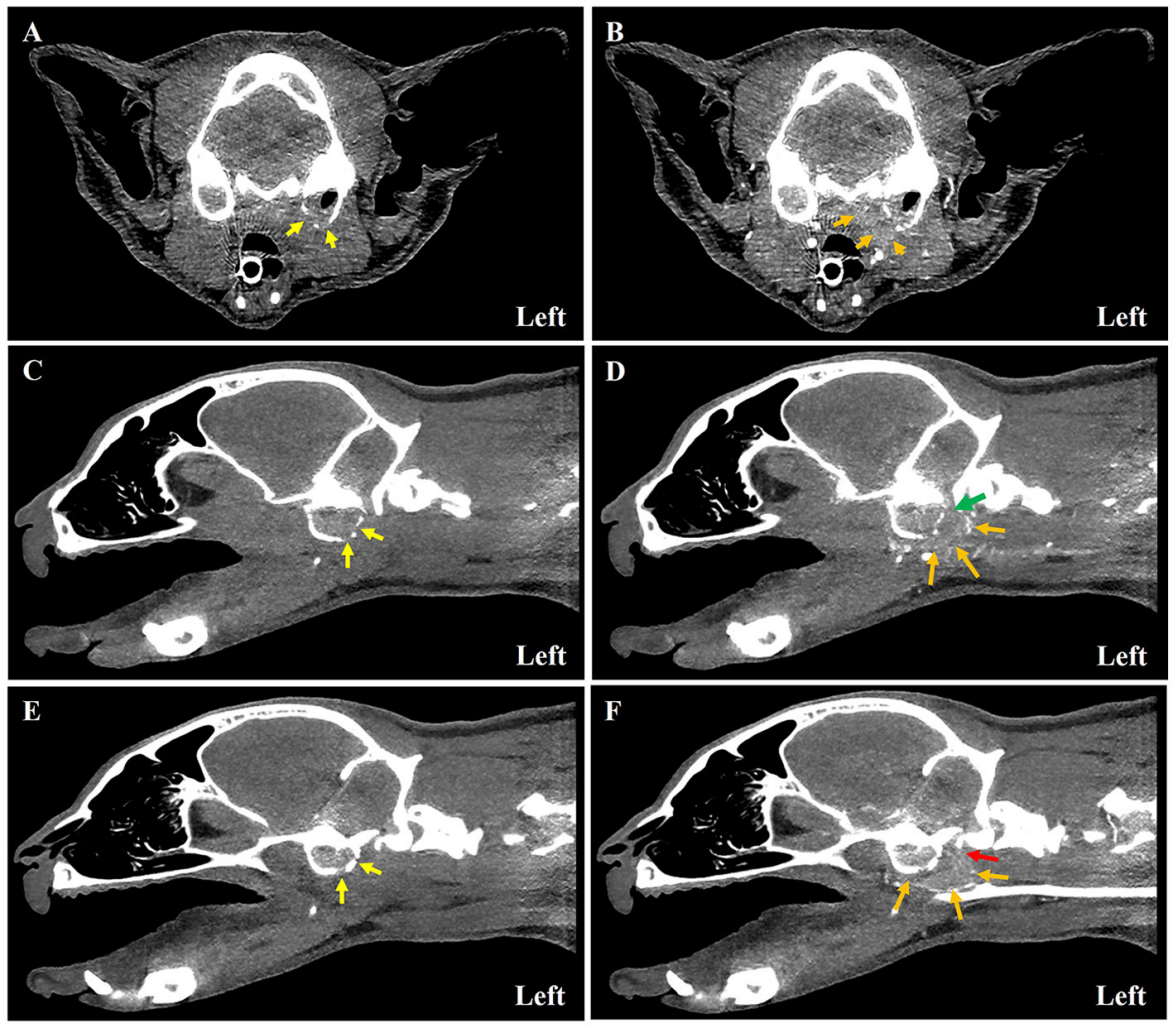
Computed tomography images of a 7-year-old American short hair cat with left-sided middle ear adenocarcinoma. Pre-contrast **(A, C, E)** and post-contrast **(B, D, F)** images of soft tissue mass around the tympanic bulla at each level. On transverse tympanic bulla level **(A, B)**, bilateral tympanic bulla was filled with soft tissue density material. The left-sided bulla was expanded, and osteolysis (yellow arrow) was marked **(A, C, E)**. The ventral margin of the bulla was disrupted, and a contrast-enhancing mass (orange arrow) around the tympanic bulla was noted **(B, D, F)**. On the sagittal view **(C–F)**, the tympano-occipital fissure (green arrow) **(C, D)** and the hypoglossal canal (red arrow) **(E, F)** were occupied with soft tissue mass.

Following the previous examination, intubation was performed, and anesthesia was maintained with isoflurane. Computed tomography (CT) (MyVet CT i3D, Woorien, Seoul, South Korea) images of the head and neck were obtained (Figure 2). CT examination demonstrated hyper-attenuating materials within the bilateral tympanic cavity. The left tympanic bulla was slightly expanded and osteolysis was noted. Postcontrast CT images revealed a contrast-enhancing mass around the left tympanic bulla. The contrast-enhancing soft tissue mass occluded the tympano-occipital fissure and hypoglossal canal. In addition, the left sternocephalicus and cleido cephalicus muscle atrophy were observed from the occipital bone to the third cervical vertebrae. The severity of atrophy of the affected muscles was approximately half of the thickness of the contralateral neck musculature.

For further visualization of the ear, we also examined the external ear canal and tympanic membrane using an otoscope (Veterinary otoscope, 67260OSA, Karl Storz, Tuttlingen, Germany). There was no definite otitis externa in the bilateral ear, but the tympanic membrane was not translucent and bulging.

Based on the examination, the cat was tentatively diagnosed with bilateral otitis media and left-side middle ear tumor which affected the jugular foramen and hypoglossal canal.

After an uneventful recovery from anesthesia, prednisolone (1 mg/kg, BID PO), amoxicillin-clavulanate (15 mg/kg, BID PO), and enrofloxacin (5 mg/kg, SID PO) were prescribed for the treatment of otitis media. However, the clinical signs deteriorated, and the patient experienced dysphagia, retching, as well as atrophy and deviation of the left-sided tongue muscle. A ventral bulla osteotomy was performed for definitive diagnosis and palliative treatment. On gross examination, tissue proliferation with inflammation was identified in the left tympanic bulla. Inflammation with tissue proliferation in the left-sided tympanic bulla using an otoscope was identified. The resected tissue sample was submitted for histopathology. Because the cat had difficulty swallowing foods, an esophagostomy tube was placed to provide nutrition.

**Figure 3:**
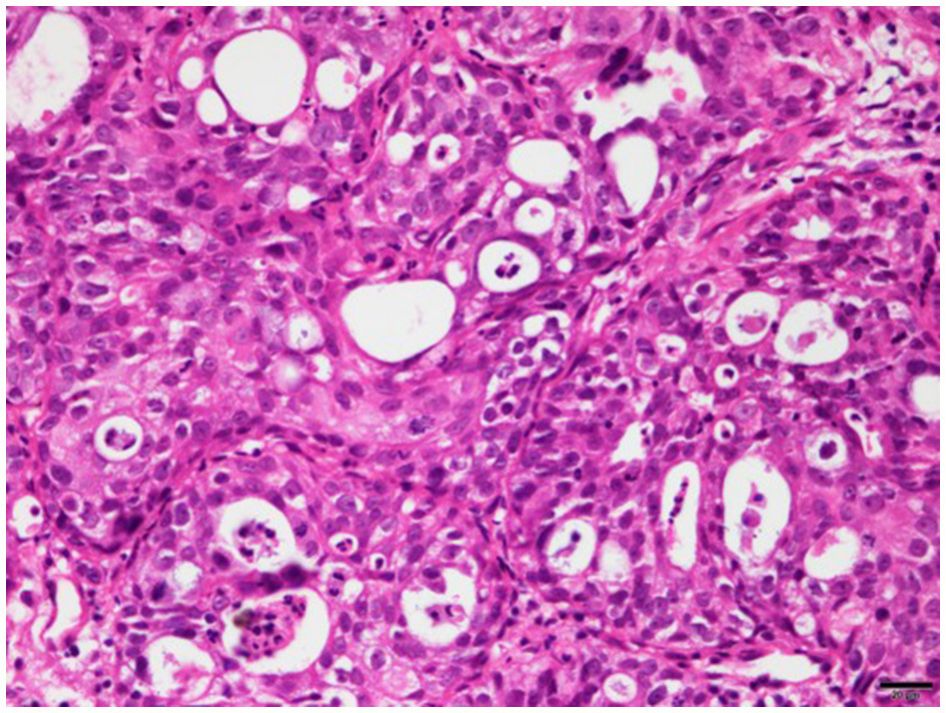
Histopathology of adenocarcinoma in the left tympanic bulla of a 7-year-old American shorthair cat. Microscopically, the mass was made up of glandular and solid pattern proliferation of polygonal to columnar epithelial cells. Neoplastic cells had abundant eosinophilic granular cytoplasm, and some cells had varying degrees of vacuolated cytoplasm. The nuclei of these cells were round to oval in shape and had one or two prominent nucleoli. These cells were moderately pleomorphic, and no mitotic figures were shown.

On histopathologic examination, invasive proliferation with unclear boundaries with surrounding tissues was identified under the mucosal layer covered with ciliary columnar epithelium. Mild-to-moderate anisokaryosis was noted, and mitotic figures were rare. The mass was diagnosed as a low-grade adenocarcinoma with mild inflammatory cells (Figure 3).

We concluded that cranial nerves IX (glossopharyngeal nerve), X (vagus nerve), XI (accessory nerve), and XII (hypoglossal nerve) and sympathetic nerve of this cat were damaged by the middle ear adenocarcinoma. The cat was treated with prednisone (2 mg/kg, BID PO), toceranib phosphate (2.5 mg/kg, three times per week PO), and gabapentin (10 mg/kg, BID PO) after a ventral bulla osteotomy. However, the clinical signs of the cat deteriorated, and the cat died due to refractory vomiting and aspiration pneumonia.

## Discussion

This case report describes a rare neurologic disease, Villaret's syndrome, first in veterinary medicine. Adenocarcinoma in the left tympanic bulla of this cat is occupied around the tympano-occipital fissure and hypoglossal canal. As a result, the adenocarcinoma compressed the LCNs through the area, damaging both the sympathetic nerves and the last four cranial nerves in this cat.

According to a previous report described in human beings (2, 5–8), Villaret's syndrome is a rare nervous syndrome that damages the last four cranial nerves and the cervical sympathetic trunk. This syndrome is mainly caused by space-occupying lesions (primary and/or metastatic tumors and aneurysms) (1, 5, 7, 8) or trauma (1, 5) in the posterior retroparotid space where cranial nerves IX, X, XI, and XII and sympathetic nerve pass. Common signs associated with this syndrome include dysphagia, dysphonia, loss of taste, trapezius and sternocleidomastoid muscle atrophy, and Horner's syndrome (1, 2, 5–9).

**Figure 4:**
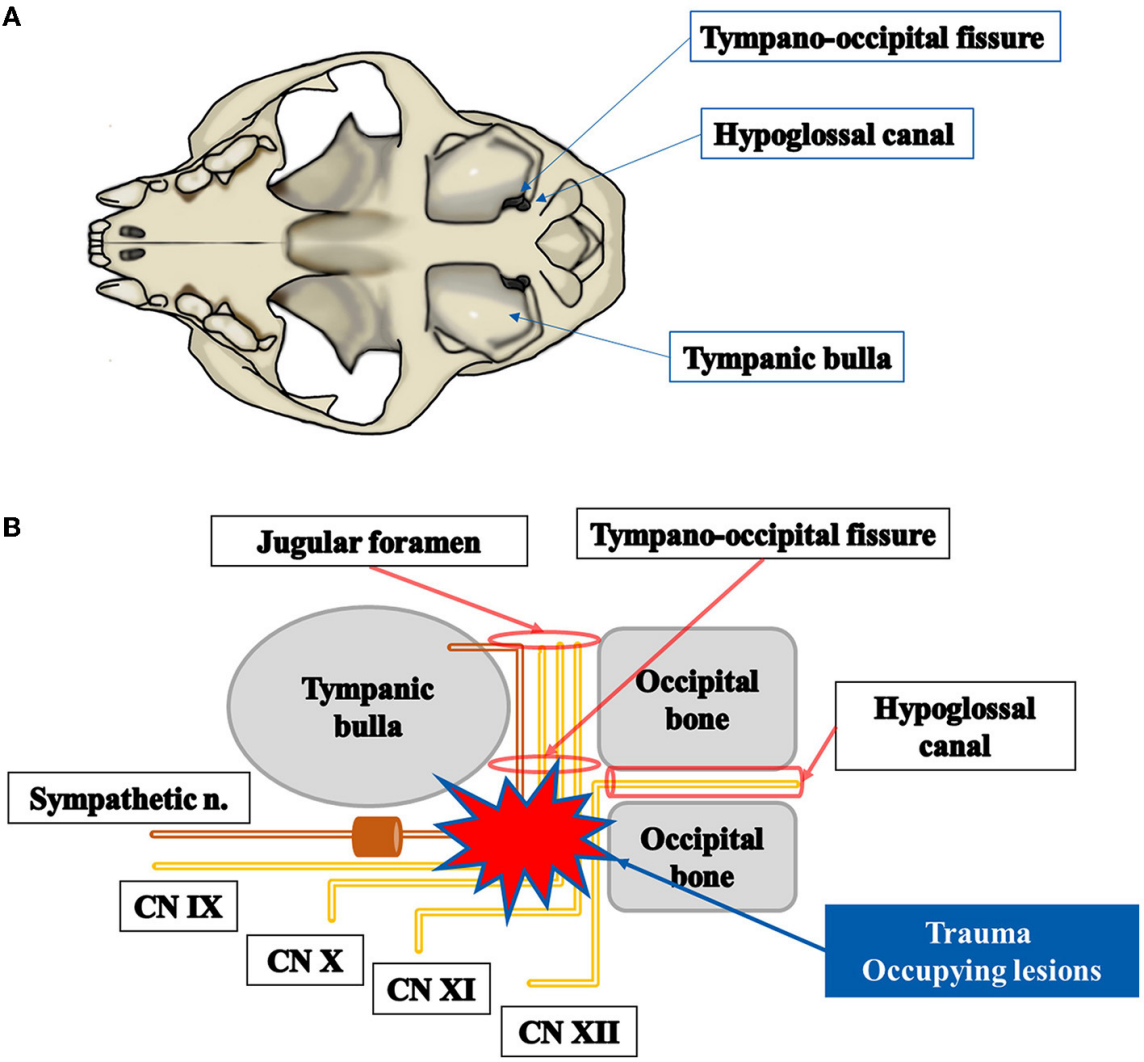
A schematic diagram of a cat's tympanic bulla, tympano-occipital fissure, and the hypoglossal canal **(A)** and nerves affected in Villaret's syndrome **(B)**. Tympano-occipital fissure and hypoglossal canal are located caudomedial to tympanic bulla **(A)**. Sympathetic nerves and lower cranial nerves such as CN IX, CN X, CN XI, and CN XII can be damaged when trauma or space-occupying lesions are present.

The cranial nerves IX, X, XI, and XII are known as LCNs or the last four cranial nerves in human medicine, and these LCN lesions cause various syndromes depending on the nerves involved, such as Villaret's syndrome, Collet–Sicard syndrome, Vernet's syndrome, or Tapia's syndrome (3, 9). The LCNs involved and clinical symptoms for each syndrome are presented in Supplementary Table 1 (2, 3, 5–13). Anatomically, the cranial nerves IX, X, and XI originate from the medulla oblongata, enter the jugular foramen, and then pass through the tympano-occipital fissure (14). The cranial nerve XII originates from the medulla oblongata and exits the cranium through the hypoglossal canal. The tympano-occipital fissure and hypoglossal canal are both anatomically located near the tympanic bulla in cats (Figure 4A). In this cat, a left-sided middle ear tumor occupied those areas and damaged the last four cranial nerves and cervical sympathetic nerves (Figure 4B). As a result, related clinical signs appeared in this cat.

Theoretically, the glossopharyngeal nerve (cranial nerve IX) functions as a sensory function in the back 1/3 of the tongue, communicates with the vagus nerve, and constitutes sensory and motor nerves in the mucous membrane and muscles of the pharynx (3, 9, 14). Therefore, when glossopharyngeal neuropathy occurs, subsequent loss of taste, reduction of gag reflex, and swallowing disorders may appear. It is difficult to determine whether this cat lost its sense of taste; however, other signs, such as reduced gag reflex and difficulty swallowing, had been observed.

Sensory and motor nerves of the vagus nerve (cranial nerve X) innervate in the pharynx, larynx, and thoracic and abdominal viscera of dogs and cats (3, 9, 14). Therefore, when vagus neuropathy occurs, various signs, such as reduction of gag reflex, dysphagia, laryngeal paralysis, inspiratory dyspnea, and regurgitation, may be present. This cat also showed reduced gag reflex, dysphagia, laryngeal paralysis, and regurgitation.

The accessory nerve (cranial nerve XI) innervates and controls the trapezius, sternocephalicus, and brachiocephalicus muscles which comprise the neck muscles (3, 9, 14). In cases of accessory nerve neuropathy, muscular atrophy can occur. In the present case, atrophy of the sternocephalicus and cleidocephalicus muscles was observed.

The hypoglossal nerve (cranial nerve XII) is mainly involved in the movement of the tongue (3, 9, 14). Therefore, in the presence of hypoglossal neuropathy, ipsilateral tongue muscle atrophy and deviation are observed, which is consistent with the cat in this report.

In general, the clinical signs of Horner's syndrome include miosis, protrusion of the third eyelid, enophthalmos, and ptosis (15). Diagnosis is mostly made by clinical signs alone, but topical phenylephrine test and, if necessary, advanced imaging studies, such as CT and magnetic resonance imaging (MRI), may be needed to localize the lesion and make a definitive diagnosis (16). Horner's syndrome is most commonly seen in lesions affecting the middle ear, and this cat had a left middle ear adenocarcinoma causing osteolysis of the tympanic bulla. Diseases affecting the tympanic bulla in cats are rare, and there are few reports of middle ear tumors (17, 18).

As described above, it is very difficult to diagnose LCNs-related syndrome because various neurological signs appear in a complex manner depending on the damaged nerves. In addition, accurate evaluation of LCNs is not easy in dogs and cats. In veterinary medicine, only one case report described the LCNs syndrome in a dog (4). A previously reported dog suffered from tympanic bulla osteoma (4), and the clinical signs of this dog were identical to our cat except for ipsilateral deviation of the tongue, a sign of hypoglossal neuropathy. The dog was diagnosed with Vernet's syndrome and Horner's syndrome caused by osteoma in the tympanic bulla and had a poor prognosis (4).

In this case, the development of clinical signs was rapid and progressive. This is suspected to be closely related to the invasion of the causative tumor as previously described (8). According to the previous reports on surgical intervention for middle ear disease in cats (19), neoplastic infiltration of the middle ear was related to the poor prognosis, and surgical intervention did not alter the course of the disease progression. Systemic chemotherapy using toceranib and prednisone was administered after a confirmed diagnosis, but it was applied only for a short period of time, and the cat died of acute dyspnea accompanied by aspiration pneumonia. This cat underwent ventral bulla osteotomy, and commonly reported side effects of this procedure include Horner's syndrome, facial nerve paralysis, and recurrence of primary lesions (19, 20). According to a recent retrospective study assessing complications associated with various types of ventral bulla osteotomy, post-operative respiratory-related complications could occur (20). However, respiratory signs were frequently observed in single-stage bilateral ventral bulla osteotomy and usually initiated with upper respiratory signs within 24 h postoperatively. In the present case, the cat recovered from the surgery without significant complications. Unfortunately, despite the administration of medication and adjunctive treatments, the neurological signs deteriorated progressively and developed aspiration pneumonia as a result of ongoing neurological dysfunction of LCNs.

This case report has several limitations. The anatomical location and pathways of the cranial nerves are complex. Because of the small size of the nerves and their non-parallel alignment with the standard imaging planes, accurate identification of specific cranial nerves deficit remains a challenging area (21, 22). While both CT and MRI can be utilized for diagnosing cranial nerve disorders, MRI stands as the best imaging modality for diagnosing cranial nerve disorders due to its superior soft tissue contrast (21). In recent years, MRI diagnostic techniques and their application have been continuously evolving in veterinary medicine and offer potential utility in the evaluation of both normal anatomical structures and pathological changes in cranial nerves (22, 23). However, in the present case, due to the rapid deterioration of clinical signs and financial constraints, additional MRI imaging was not obtained. In addition, the exact cause of death has not been definitively determined in this cat. Although it is not possible to entirely rule out the effects of anesthesia and surgery as the underlying cause of the cat's respiratory distress, the cat recovered well without significant complications after the surgery. Therefore, considering the progressive worsening of neurological signs and the development of aspiration pneumonia due to persistent retching, it is suggested that the death of this cat was related to the progression of the primary disease.

In conclusion, to the best of our knowledge, this is the first case report describing Villaret's syndrome in a cat. In cats with neuropathy associated with LCN syndromes, middle ear disease should be considered a differential diagnosis. In addition, accurate anatomical knowledge and physiological understanding of the area through which the LCNs pass, as well as awareness of these neurological diseases, would be helpful for the diagnosis of similar neuropathies.

## Data availability statement

The original contributions presented in the study are included in the article/Supplementary material. Further inquiries can be directed to the corresponding author.

## Ethics statement

Written informed consent was obtained from the participant/patient(s) for the publication of this case report.

## Author contributions

D-JK and M-HK wrote the manuscript. D-JK, W-KP, S-YK, and D-HS assisted in the supervision of the clinical management of this case and contributed to the conception of the case report. H-MP and M-HK supervised the clinical management of this case and revised the manuscript. S-YK assisted in the cytologic evaluation and histopathology review of this case. D-HS assisted anesthetic process and surgery. All authors critically reviewed and approved the final version of the manuscript.
