# Supplementary material for: Case report: Villaret's syndrome caused by middle ear adenocarcinoma in a cat
Source: Front Vet Sci. 2023 Jul 27;10:1225567. doi: 10.3389/fvets.2023.1225567 (PMC10413872; doi:10.3389/fvets.2023.1225567)
Supplement: Supplementary file 1 [file Table_1.docx]

**Table**

**Table 1.** Lower cranial nerve syndromes described in human medicine

| Syndrome | Affected nerves | Signs |
| --- | --- | --- |
| Villaret’s syndrome  (2, 3, 5-9) | CN IX, X, XI, XII, Sympathetic nerve | Taste disorder in posterior third of tongue  Loss of sensation from soft palate and posterior third of tongue  Reduction of gag reflex  Dysphonia  Atrophy of trapezius and sternocleidomastoid muscle  Ipsilateral deviation of the tongue  Horner’s syndrome |
| Collet-Sicard syndrome  (3, 9-11) | CN IX, X, XI, XII | Taste disorder in posterior third of tongue  Loss of sensation from soft palate and posterior third of tongue  Reduction of gag reflex  Dysphonia  Atrophy of trapezius and sternocleidomastoid muscle  Ipsilateral deviation of the tongue |
| Vernet syndrome  (3, 9, 12) | CN IX, X, XI | Taste disorder in posterior third of tongue  Loss of sensation from soft palate, posterior third of tongue  Reduction of gag reflex  Dysphonia  Atrophy of trapezius and sternocleidomastoid muscle |
| Tapia’s syndrome  (3, 9, 13) | CN X, XII | Dysphagia  Dysphonia  Ipsilateral deviation of the tongue |

CN, cranial nerve
